# Impact of health insurance equity on poverty vulnerability: evidence from urban–rural health insurance integration in rural China

**DOI:** 10.3389/fpubh.2023.1328265

**Published:** 2023-11-30

**Authors:** Zhipeng Li, Yuqian Chen, Jing Ding

**Affiliations:** ^1^Qu Qiubai School of Government, Changzhou University, Changzhou, China; ^2^School of Economics and Management, Shanghai University of Political Science and Law, Shanghai, China; ^3^School of Public Economics and Administration, Shanghai University of Finance and Economics, Shanghai, China

**Keywords:** urban–rural health insurance integration, health insurance equity, poverty vulnerability, rural China, healthcare utilization, poverty due to illness

## Abstract

**Background:**

In 2016, the Chinese government introduced an integration reform of the health insurance system with the aim to enhance equity in healthcare coverage and reduce disparities between urban and rural sectors. The gradual introduction of the policy integrating urban and rural medical insurance in pilot cities provides an opportunity to evaluate the policy impact. This study attempts to assess the policy impact of urban–rural health insurance integration on the chronic poverty of rural residents and to analyze the mechanisms.

**Method:**

Based on the four waves of data from the China Health and Retirement Longitudinal Study (CHARLS) conducted in 2011, 2013, 2015, and 2018, we employed a staggered difference-in-differences (staggered DID) model to assess the impact of integrating urban–rural health insurance on poverty vulnerability among rural inhabitants and a mediation model to analyze the mechanism channel of the policy impact.

**Results:**

(1) Baseline regression analysis revealed that the urban–rural health insurance integration significantly reduced the poverty vulnerability of rural residents by 6.32% (*p* < 0.01). The one health insurance system with one unified scheme of contributions and benefits package (OSOS, 6.27%, *p* < 0.01) is more effective than the transitional one health insurance system with multiple schemes (OSMS, 3.25%, *p* < 0.01). (2) The heterogeneity analysis results showed that the urban–rural health insurance integration had a more significant impact on vulnerable groups with relatively poor health (7.84%, *p* < 0.1) than those with fairly good health (6.07%, *p* < 0.01), and it also significantly reduced the poverty vulnerability of the group with chronic diseases by 9.59% (*p* < 0.01). The integration policy can significantly reduce the poverty vulnerability of the low consumption and low medical expenditure groups by 8.6% (*p* < 0.01) and 7.64% (*p* < 0.01), respectively, compared to their counterparts. (3) The mechanism analysis results showed that the urban–rural health insurance integration can partially enhance labor supply (14.23%, *p* < 0.01) and physical examinations (6.28%, *p* < 0.01). The indirect effects of labor supply and physical examination in reducing poverty vulnerability are 0.14%, 0.13% respectively.

**Conclusion:**

The urban–rural health insurance integration policy significantly reduced poverty vulnerability, and the OSOS is more effective than the OSMS. The urban–rural health insurance integration policy can significantly reduce poverty vulnerability for low consumption and poor health groups. Labor supply and physical examination are indirect channels of the impact. Both channels potentially increase rural household income and expectations of investment in human health capital to achieve the policy objective of eliminating chronic poverty.

## 1 Introduction

China has achieved remarkable success in its anti-poverty strategies and social security policies. According to the international poverty standards set by the World Bank, China’s efforts in reducing poverty accounted for over 70% of the global poverty reduction, significantly advancing the progress of global poverty alleviation (1). The financial risks of pursuing medical treatment for severe illnesses can still devastate low- and middle-income families. On the one hand, as of 2015, China has achieved an introductory medical insurance coverage rate exceeding 95% of its population of 1.336 billion (2), and the medical insurance coverage rate remained stable at more than 95% in 2022, with the number of participants reaching 1.346 billion (3). The basic medical security system for universal coverage has been essentially completed. On the other hand, according to official poverty statistics from 2018, over 42% of registered poor households in China experienced poverty due to illness (4), and the proportion of poverty due to illness remained at 40% in 2022 (5). This has resulted in the coexistence of “broad coverage” basic medical insurance and “high proportion” illness-induced poverty.

High medical expenses will transform uncertain health risks into economic risks affecting family welfare. The prepayment pooling mechanism established by health insurance can provide social protection. Health insurance with comprehensive coverage and sufficient safeguards is one of the most crucial measures to combat poverty (6). However, the varying arrangements of health insurance systems for different population groups may diminish the anti-poverty effect of health insurance and result in inequity. China has witnessed a remarkable expansion in social health insurance coverage, with the establishment of the New Rural Cooperative Medical System (NCMS) and Urban Residents’ Basic Medical Insurance (URBMI) in 2003 and 2007, respectively, catering to rural residents and urban non-employed individuals.

The utilization of outpatient and inpatient services has increased under the NCMS (7) while the cost of deliveries has decreased (8). However, there has been no reduction in overall out-of-pocket payments, and the health status of rural residents enrolled in the NCMS remains the same (9). There are indications of moral hazards on the supply side that the program has increased ownership of expensive equipment among central township health centers (10) and had no impact on cost per case. Additionally, participants in the NCMS receive relatively lower reimbursement ratios than their counterparts in the URBMI due to constrained funding (11). Although designed to protect against economic risk from inpatient care costs, the limited protective effect of the NCMS on medical impoverishment is primarily due to expensive outpatient services for chronic conditions (12).

The health outcomes of individuals covered by URBMI were significantly superior to those without insurance. The availability and quality of inpatient care for enrollees have improved, both while avoiding additional costs (13). The URBMI is designed as an equitable financing policy, with premiums not varying based on income or education levels. Low-income families express higher satisfaction levels with the URBMI (14), although beneficiaries from higher-income groups tend to benefit more than those from lower-income groups. In other words, the health insurance fund (primarily composed of government subsidies) intended to support vulnerable populations has disproportionately benefited wealthier individuals (15).

Inequitable access to healthcare and financial protection for rural residents results from the fragmented social health insurance schemes between urban and rural regions (16). First, rural residents face lower actual reimbursement rates when seeking better quality medical services in advanced medical institutions in cities than their urban counterparts, resulting in heavier medical economic burdens. Second, the financing of the NCMS at the county level significantly weakens the portability of health insurance and the flow of rural residents seeking better job opportunities between counties and cities. Third, segmented health insurance systems have caused inefficiencies such as enrollee repeated participation that increase unnecessary operating costs and fiscal burden while distorting economic resource allocation and information sharing. To address healthcare inequality derived from the fragmentation of social health insurance between urban and rural areas, China implemented reforms to merge the NCMS with the URBMI, forming the Urban–Rural Resident Basic Medical Insurance (URRBMI). The URRBMI policy was officially implemented in 2016, but some provinces/cities began pilot policies around 2009.

The URRBMI significantly enhances inpatient care utilization among rural residents, particularly in the middle-aged and older adult groups, while demonstrating limited impact on improving health outcomes (17). The URRBMI notably increases consumption among vulnerable households with lower wealth or higher health risks by directly reducing medical expenses and indirectly influencing precautionary savings (18). The URRBMI expands the income group of rural residents, reduces out-of-pocket payments, improves the financial protection provided by basic medical insurance, benefits more low-income rural residents, and further enhances the overall health performance of rural communities. Implementing the URRBMI policy raises reimbursement rates and significantly improves financial protection and health performance. This is especially beneficial for low-income individuals within rural areas (19).

The research purpose of this study as follows. First, some pieces of literature have discussed the impact of the URRBMI on healthcare utilization and consumption. However, few studies have focused on the causal relationship between the URRBMI and persistent poverty. Our study attempts to fill this literature gap by systematically evaluating the impact of the URRBMI on rural residents’ poverty vulnerability. Second, we conducted an empirical study with a more rigorous research design, which will make the results of policy evaluation more reliable and robust. The urban and rural health insurance integration in China was gradually carried out in local provinces and cities from 2009 to 2020, which allowed us to employ a staggered difference-in-differences (staggered DID) mode to evaluate the policy effect of URRBMI through the empirical strategy of the quasi-natural experiment. We carefully checked the policy implementation time announced on local official websites and combined it with four waves of data from the China Health and Retirement Longitudinal Study (CHARLS) for 2011, 2013, 2015, and 2018. Third, we used the mediator effect model to explore the mechanism of the URRBMI in reducing the possibility of poverty in the future by promoting physical examinations and increasing labor supply.

## 2 Institutional background

China has been unswervingly committed to the public policy practice of universal healthcare coverage (UHC) to protect all citizens, especially the impoverished population, from being excluded from the healthcare system due to economic risk. Since the establishment of the Urban Employee Basic Medical Insurance (UEBMI) for urban workers in 1998, the Chinese central government has unveiled a long-term plan for establishing multiple social health insurance schemes. It has expanded the coverage of the overall population. The New Rural Cooperative Medical System (NCMS) for rural residents and Urban Residents’ Basic Medical Insurance (URBMI) for urban non-employed residents were established in 2003 and 2007, respectively. The overall participation rate of basic medical insurance was only 22.1% in 2003 (49.4% in urban areas and 12.6% in rural areas). With the continuous improvement of the basic medical insurance system, the overall coverage rate rose to 87.1% in 2008 (92.5% in urban areas and 71.9% in rural areas) (20).

China implemented a milestone healthcare reform in 2009 to provide affordable and equitable primary healthcare for all. This reform proposed a notable idea for integrating basic medical insurance to address health inequalities across urban–rural regions and operation inefficiencies. Since 2009, several provinces and cities, particularly those in the economically developed eastern coastal areas, have gradually initiated pilot policies for integrating urban–rural health insurance integration with the guiding principles of the healthcare reform policy document. In 2016, the State Council of China issued an official policy document on integrating the basic medical insurance system for the NCMS and URBMI, establishing six unified policy implementation principles (i.e., unified coverage, unified financing policy, unified benefits packages, unified catalog of health services and drugs, unified designated medical institutions, and unified fund management) to promote the integration process. The central government formulates the framework and policies uniformly for urban–rural health insurance integration, while local governments are responsible for implementing these policies. Before the State Council’s document issuance, nine provinces, including Tianjin, Shanghai, Zhejiang, Shandong, Guangdong, Chongqing, Ningxia, Qinghai, and Xinjiang, had already promoted integration efforts by establishing a unified medical insurance system for both urban and rural residents.

Three crucial policy changes can enhance the policy effectiveness of health insurance poverty alleviation. First, the URBMI and NCMS are administered by the Chinese Ministry of Human Resources and Social Security (MoHRSS) and China’s National Health Commission (NHC, previously China’s Ministry of Health), respectively. After implementing the integration policy, the NCMS and URBMI were uniformly administered by the MoHRSS for the Urban and Rural Residents’ Basic Medical Insurance (URRBMI). Table 1 provides concrete details about the health insurance schemes of the URBMI, NCMS, and URRBMI. There are no rural or urban identity differences (i.e., the hukou requirement) for participating in the URRBMI. The unification of administration has relieved the waste of human resources and financial and economic burden, while information system sharing can effectively regulate duplicate health insurance participation problems. In 2018, China established the National Healthcare Security Administration (NHSA), which was in charge of the URRBMI (merged from the NCMS and URBMI) and UEBMI. Second, the financing levels of the URRBMI have been upgraded from the county to the municipal level. This dramatically improves health insurance portability and expands the list of drugs and healthcare services. When rural residents go from towns or counties to local cities for healthcare in high-level healthcare service institutions (i.e., tertiary hospitals in China), they gain higher reimbursement than with the NCMS. The expansion fund pooling of the URRBMI has strengthened the capacity to prevent economic risks. Third, a differential premium payment standards and benefits package could be adopted temporarily in areas where there is a significant difference in individual contributions standards between the URBMI and NCMS before implementing the integration policy. The central government allows local governments to gradually unify the scope of healthcare services and reimbursement standards, transitioning over 2–3 years. After urban–rural health insurance integration, the actual *per capita* contribution and benefits package should not be lower than the current level. In the policy process promoting urban–rural health insurance integration, there are differences in the policies adopted by local governments. Some cities adopt an approach of one system with one scheme of contribution and benefits package (i.e., the OSOS), which directly unifies the financing and security benefits of the NCMS and URBMI in one step. Some cities adopt another approach of one system with multiple schemes (i.e., the OSMS), which links premium payment standards with reimbursement standards. In general, the OSMS offers three choices of health insurance scheme (scheme I, II, and III). Scheme I corresponds to a lower contribution and benefits package, and is close to the original standard for the NCMS. Scheme II corresponds to a moderate contribution and benefits package, and is close to the initial standard for the URBMI. Compared to the formers, scheme III corresponds to a higher level of contribution and benefits package, and is lower than the standard for the UEBMI. We use Qiqihaer City in Heilongjiang province in China as an example to illustrate the details of health insurance schemes differences in the details of the three health insurance schemes offered by the OSMS (Appendix Tables A1, A2 in Supplementary material). Although there are no differences in the benefits package of the OSOS for urban and rural residents, the relatively high level of premium payment of funding contributions is likely to exclude the participation of low-income rural families from the health insurance system, which means there is a potential possibility to reduce the coverage rate of health insurance for rural residents. The OSMS provides multiple health insurance scheme selections for participants, who can choose corresponding health insurance schemes based on their economic capabilities, ensuring the accessibility of health insurance to low-income rural families. Moreover, the transition plan of the OSMS can alleviate the critical pressure on the finance funding of health insurance in the initial implementation stage of the urban–rural health insurance. The problem of the OSMS is that the NCMS and URBMI formally integrate a health insurance system. Still, it does not entirely change the substantive multi-layer policy institutional arrangement to promote health insurance equity. When the equity of health insurance is improved, what are the policy effect differences between the OSOS and OSMS on long-term poverty? This question needs to be answered through rigorous empirical research. The gradual implementation of the pilot policy process for the urban–rural health insurance integration provides a pivotal opportunity to identify policy effects through the research design of quasi-natural experiments. To assess the impact of this integration on poverty vulnerability, we utilize the staggered DID econometric model and analyze potential impact mechanisms.

**Table 1:** Introduction to the institutional background of basic social medical insurance systems for urban and rural residents in China (URBMI, NCMS, and URRBMI).

Scheme	URBMI	NCMS	URRBMI
Coverage eligibility	Urban non-working residents (including infants, children, and various types of students on campus)	Rural residents	Rural and urban residents not covered by the UEBMI
Benefits package	Mainly for inpatients and a unified reimbursement ratio of specific diseases for outpatients	Mainly for inpatients and a unified reimbursement ratio of specific diseases for outpatients	Mainly for inpatients and a unified reimbursement ratio of specific diseases for outpatients
The level of fund pooling	Municipal level	County level	Municipal level
Financing model	Individual contribution + Government subsidy	Individual contribution + Government subsidy	Individual contribution + Government subsidy
Reimbursable ratio and list of drugs and medical services	Higher and wider	Lower and narrower	Upgrade for URBMI standards
Implementation time	2007	2003	Gradually, pilot cities from 2009; official policy documents implemented in 2016
Administration	MoHRSS	NHC	MoHRSS from the integration, NHSA from 2018

Based on previous vital studies (19, 21, 22), we summarize the details of the institutional background for China’s basic social medical insurance systems for urban and rural residents and supplement the reform of management institutions after 2018.URBMI, Urban Residents’ Basic Medical Insurance; NCMS, New Rural Cooperative Medical System; URRBMI, Urban and Rural Residents’ Basic Medical Insurance; UEBMI, Urban Employee Basic Medical Insurance; MoHRSS, Chinese Ministry of Human Resources and Social Security; NHSA, National Healthcare Security Administration; NHC, National Health Commission.

### 2.1 Data and method

**Figure 1:**
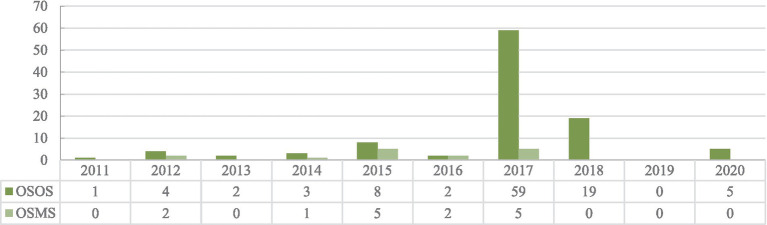
Accumulated number distribution of the Chinese cities implementing the policy of urban–rural health insurance integration for both the OSOS and OSMS. Based on the CHARLS sample, time information for the implementation of urban–rural medical insurance integration in cities is collated according to the official website document of the local government department responsible for administering the health insurance.

We have compiled official policy regulations and documents released by the administration responsible for health insurance in local governments and collected the policy implementation time of urban–rural health insurance integration to form the city-level policy dataset (see Figure 1). There are 118 cities that implemented integration policies (103 cities for the OSOS and 15 cities for the OSMS) from 2011 to 2018, and we matched the city-level dataset information with individual-level microdata to form the available dataset for policy evaluation.

**Figure 2:**
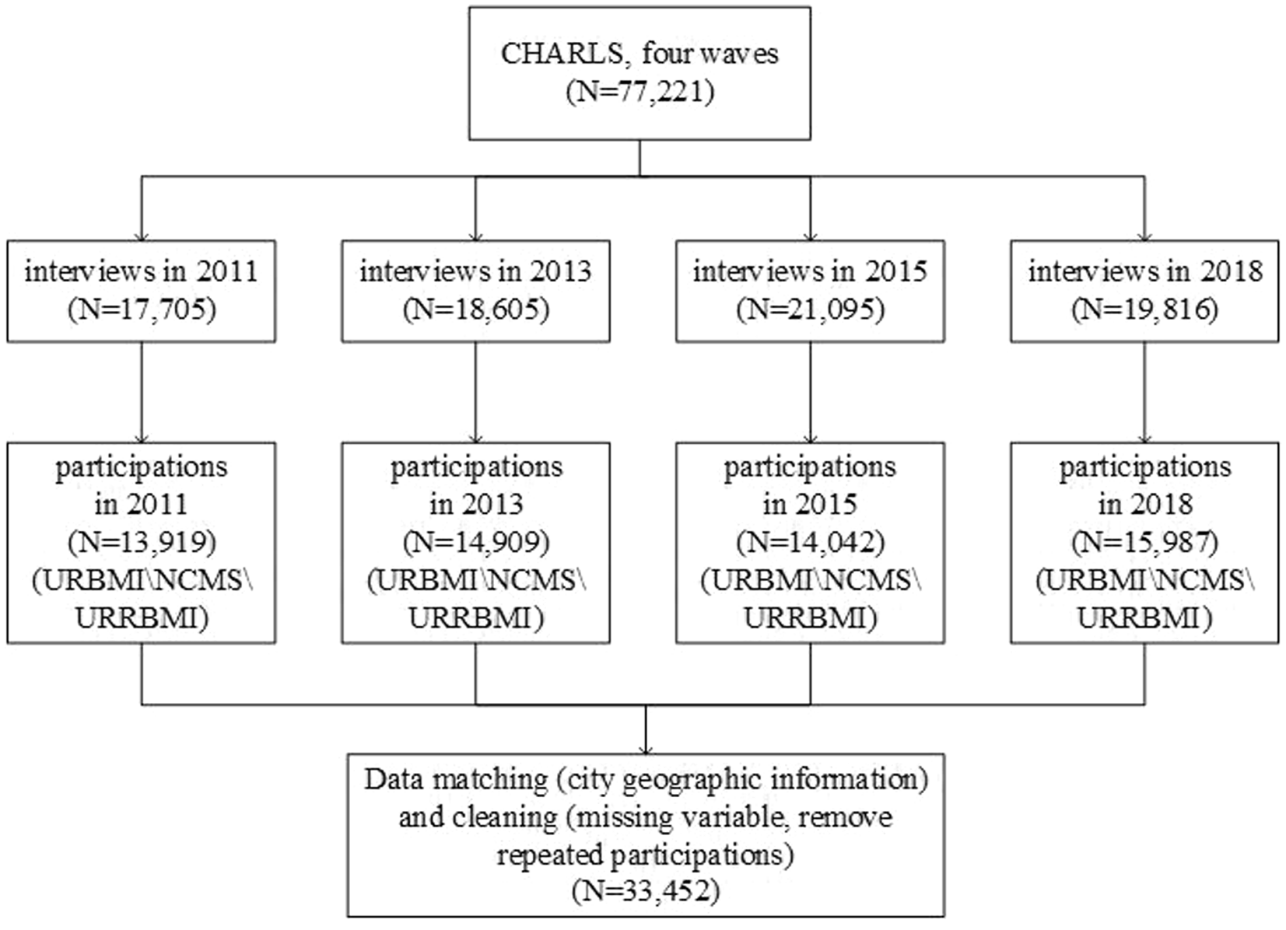
Flowchart of population selection.

The China Health and Retirement Longitudinal Study (CHARLS) is a nationally comprehensive individual-level dataset, which is a large-scale interdisciplinary survey project led by the National Development Research Institute of Peking University, jointly executed by the China Social Sciences Survey Center of Peking University and the Youth League Committee of Peking University. The project aims to collect high-quality microdata representing Chinese residents aged 45 and older. The national baseline survey began in 2011, and follow-up surveys were conducted every two to three years, covering 28 provinces (municipalities, autonomous regions), 150 county-level units, and 450 community (village) units nationwide. As of the completion of the fourth wave nationwide follow-up survey in 2018, the sample covered a total of about 19,000 respondents from 12,400 households. The questionnaire included: demographic information, family structure, economic relations, health examination status, medical service utilization, medical insurance, work and labor supply, social security income, consumption, assets, and community information. The CHARLS has been widely recognized and applied in academic quantitative research due to its high-quality sample representation and data collection response rate. Our paper uses the four waves of the CHARLS (2011, 2013, 2015, and 2018) for the following considerations: first, the policy of urban-rural health insurance integration is implemented gradually in different provinces and cities. The CHARLS releases important geographical information about where the cities of individual household samples are located. Second, the respondents to the CHARLS are mainly aged 45 and older, with more demand for healthcare. The CHARLS includes comprehensive information on health insurance, healthcare services, and medical cost expenditures. Third, we can adopt a rigorous research design based on the data structure of four waves of the CHARLS for evaluating the dynamic results of urban–rural health insurance integration on poverty vulnerability and ensuring the robustness of the estimated impacts of policy effects. Our research aims to explore the integration policy effect for rural residents who participated in the URRBMI merged from the NCMS and URBMI. We removed samples with urban resident hukou registration, duplicate health insurance coverage, participating in the UEBMI or commercial health insurance, and being uninsured. The estimation bias errors caused by the staggered DID model have aroused extensive discussion in the academic community (23–26). We can relieve estimation bias errors through rigorous research design, including as many samples as possible that have always been in the control group during the sample period and minimizing the number of samples in the treatment group since the beginning of the sample period (25). We have removed samples of the policy implementation time before 2011, including Jiaxing (2003), Shenzhen (2004), Foshan (2007), Chongqing (2009), Chengdu (2009), Jiangmen (2010), and Tianjin (2010). After data processing, the final sample size for our research was 33,452 observations from 116 prefecture-level cities in 26 provinces for 4 years (see Figure 2). The control variable data at the city and provincial levels were collected from the China City Statistical Yearbook and the National Bureau of Statistics. We matched macro data at the city and provincial levels with individual microdata from the CHARLS based on the year and city geographic information.

### 2.2 Description of variables

#### 2.2.1 Dependent variable

To gain a comprehensive understanding of chronic poverty, we leverage the concept of poverty vulnerability. Within the framework of poverty alleviation, it is generally defined as the ex-ante risk (i.e., adverse shocks, serious illness, lost job, bad harvest) that causes a household that is currently non-poor to fall below the poverty line or a household that is currently poor to remain in poverty (27–30). Poverty vulnerability could be formally defined as the probability that the welfare (i.e., income) of a household *i* at time *t* will be below the poverty line at time *t* + 1.


(1) $$V_{i,t}=\mathrm{Pr}\left(\text{incom}e_{i,t+1}\leq z\right)$$

where $\text{incom}e_{i,t+1}$ is *per capita* household income and *z* is the official income poverty line. The current poverty standard means that the living standard of rural residents is less than 2,300 yuan *per capita* per year at the 2010 price in China, corresponding to the CHARLS sample survey timings (2,536 yuan for 2011, 2,736 yuan for 2013, 2,855 yuan for 2015, and 2,995 yuan for 2018).

To estimate parameters and compute poverty vulnerability, we deploy a three-step feasible generalized least squares (FGLS) procedure (27, 31). An in-depth exploration of the estimation methodologies and calculations can be found in Appendix A in Supplementary material.

#### 2.2.2 The key independent variable

The policy treatment variable of urban–rural health insurance integration is the key independent variable. We introduce three types of policy variables to identify the integration policy effects. First, the policy treatment variable, $\text{Dpolic}y_{\text{ist}}$ , is set to 1 as individual I located in city s which implemented the urban–rural health insurance integration policy in the year t, and 0 for otherwise. Second, the policy treatment variable of $\text{Dpolic}y_{\text{ist},\text{OSOS}}$ indicates that the city adopted a unified policy with one scheme. The policy treatment variable of $\text{Dpolic}y_{\text{ist},\text{OSMS}}$ indicates that the city adopted differentiated policies with multiple schemes. The control group refers to the samples that did not implement the urban–rural health insurance integration policy during the sample time, and is set to the baseline control group to avoid the dummy variable trap in the regression. Third, in order to estimate the dynamic policy effects of the urban-rural health insurance integration, multiple policy periods dummy variables are introduced in our study, corresponding to the seven periods consisting of the occurrence of integration policy, before three periods and after three periods. As an example, assume that the year of policy implementation in a certain city is 2013, the definition of $\text{Dpolic}y_{\text{is},t-1}$ , “the integration policy implementation before one period,” is set to 1 in the first round of the CHARLS 2011 (national baseline survey) and 0 for otherwise. The definition of $\text{Dpolic}y_{\text{is},t+2}$ , “the integration policy implementation after two period,” in the fourth round of the CHARLS 2018 survey (the third national follow-up survey) is 1, and 0 for otherwise.

#### 2.2.3 Control variables

To obtain reliable and consistent estimation of the rural–urban healthcare integration policy effects, we controlled for a range of variables, including individual and household demographic characteristics, such as age, gender, marital status, education level, party membership, ethnic minority, self-assessed health, family size, household income, and eligibility for social assistance. We also controlled the variables as to whether sewer systems and asphalt roads have been built in a community or village, the *per capita* gross domestic product (GDP) in a city, and the number of medical institution beds per 10,000 people in a province. These control variables, which may potentially affect the implementation of integration policies, represent the level of community infrastructure, the level of urban economic development, and the distribution of medical resources at the provincial level, respectively. We provide more details: the definition of the variables and the descriptive statistics of the sample in the baseline regression are provided in Tables 2, 3, respectively.

**Table 2:** Definition of variables.

Variable	Definition
*Dependent variables*	
poverty_vulnerability_ist_	The probability that *per capita* household income at time t will be below the poverty line at time t + 1. 1 for the probability of poverty vulnerability exceeds 0.5, 0 for otherwise.
*Dpolicy*_ist_	1 for the implementation of the policy of urban–rural health insurance integration, 0 for otherwise.
*Dpolicy*_ist,OSOS_	1 for the implementation of the policy of urban–rural health insurance integration with OSOS, 0 for otherwise.
*Dpolicy*_ist,OSMS_	1 for the implementation of the policy of urban–rural health insurance integration with OSMS, 0 for otherwise.
*Independent variables*	
Age	The age of respondents in the year of the data survey.
Gender	1 for female, 0 for male.
Marital status	1 for married or cohabiting, 0 for otherwise.
Education level	The years of education.
Party membership	1 for party membership, 0 for otherwise.
Ethnic minority	1 for ethnic minority, 0 for otherwise.
Self-assessed health	1 for self-assessed health level being above good, 0 for otherwise.
Family size	The number of people living together in a household.
ln(household income)	Total income earned by all family members (in logarithmic form).
Eligibility for social assistance	1 for the family being eligible for government subsidies for officially recognized low-income families, 0 for otherwise.
Asphalt roads	1 for asphalt roads having been built in a community or village, 0 for otherwise.
Sewer system	1 for sewer systems having been built in a community or village, 0 for otherwise.
ln(*per capita* GDP)	*Per capita* gross domestic product (GDP, unit: 100 million yuan) of a city (in logarithmic form).
Medical beds	The number of medical beds per 10,000 people in a province.

We set years of schooling for the variable of education level as follows: (1) No formal education (illiterate) for 1.5; (2) Did not finish primary school but capable of reading and/or writing for 3; (3) Elementary school (Sishu/home school) for 6; (4) Middle school for 9; (5) High school (vocational school) for 12; (6) Two-/Three-year college/Associate degree for 15; (7) Four-year college/Bachelor’s degree for 16; (8) Master’s degree for 18.5; and (9) Doctoral degree/Ph.D. for 22. The variable of self-assessed health is set to 1 for Excellent, Very good, and Good, and 0 for Fair, Poor, and Very poor.

**Table 3:** Summary statistics.

Variable	*N*	Mean	S.D.	Min	Max
*Dependent variables*					
poverty_vulnerability_ist_	33,452	0.298	0.457	0	1
*Dpolicy*_ist_	33,452	0.338	0.473	0	1
*Dpolicy*_ist,OSOS_	33,452	0.285	0.452	0	1
*Dpolicy*_ist,OSMS_	33,452	0.0427	0.202	0	1
*Independent variables*					
Age	33,452	60.31	9.747	35	97
Gender	33,452	0.52	0.5	0	1
Marital status	33,452	0.879	0.326	0	1
Education level	33,452	5.025	3.319	1.5	16
Party membership	33,452	0.075	0.263	0	1
Ethnic minority	33,452	0.066	0.247	0	1
Self-assessed health	33,452	0.288	0.453	0	1
Family size	33,452	2.965	1.597	1	16
ln(household income)	33,452	9.264	1.621	0	15.4
Eligibility for social assistance	33,452	0.113	0.316	0	1
Asphalt roads	33,452	0.598	0.49	0	1
Sewer system	33,452	0.186	0.389	0	1
ln(*per capita* GDP)	33,452	10.49	0.593	8.8	12.2
Medical beds	33,452	49.22	10.48	27.7	75.5

The summary statistics reported in Table 3 are based on the baseline regression sample.

### 2.3 Empirical strategy

The urban–rural health insurance integration policy pilot is gradually being implemented in various cities, which gives us the opportunity for a quasi-natural experiment. We employed a staggered DID model as an empirical strategy to identify the integration policy effect on poverty vulnerability of rural residents through the overlapping DID model, as shown in Eqs. (2, 3).


(2) $$\begin{array}{l} \text{Poverty}:\text{vulnerablit}y_{\text{ist}}=\beta_{0}+\beta_{1}\text{Dpolic}y_{\text{ist}}\\ \;+\gamma X_{\text{ist}}+\mu_{i}+\lambda_{t}+\varepsilon_{\text{ist}} \end{array}$$

(3) $$\begin{array}{l} \text{Poverty}:\text{vulnerablit}y_{\text{ist}}=\beta_{0}+\beta_{2}\text{Dpolic}y_{\text{ist},\text{OSOS}}+\\ \;+\beta_{3}\text{Dpolic}y_{\text{ist},\text{OSMS}}+\gamma X_{\text{ist}}\\ \;+\mu_{i}+\lambda_{t}+\varepsilon_{\text{ist}} \end{array}$$

(4) $$\begin{array}{l} \text{Poverty}\_\text{vulnerablit}y_{\text{ist}}=\beta_{0}+\beta_{t-t_{0}=-3}^{pre}\text{Dpolic}y_{\text{is},t-t_{0}<-3}\\ \;+\sum_{t-t_{0}=-1}^{-3}\beta_{t-t_{0}}^{pre}\text{Dpolic}y_{\text{is},t-t_{0}}\\ \;+\sum_{t-t_{0}=1}^{3}\beta_{t-t_{0}}^{\text{post}}\text{Dpolic}y_{\text{is},t-t_{0}}\\ \;+\beta_{t-t_{0}=3}^{\text{post}}\text{Dpolic}y_{\text{is},t-t_{0}>3}\\ \;+\gamma X_{ist}+\mu_{i}+\lambda_{t}+\varepsilon_{\text{ist}} \end{array}$$

$\text{Poverty}:\text{vulnerablit}y_{\text{ist}}$ represents the dependent variable of the probability of a household i falling below the poverty line in prefectural city s in *t* + 1 year. $\text{Dpolic}y_{\text{ist}}$ ( $\text{Dpolic}y_{\text{ist},\text{OSOS}}$ , $\text{Dpolic}y_{\text{ist},\text{OSMS}}$ ) is the key independent variable that the city s of household i has implemented an integration policy in year t. $X_{\text{ist}}$ is a bundle of control variables. $\mu_{i}$ is the fixed effects at the individual level and $\lambda_{t}$ is time-fixed effect. $\varepsilon_{\text{ist}}$ is the error term. An important contribution of our study is to compare the differences in policy effects between the OSOS and OSMS. This also introduces the individual-level self-selection problem into the estimation model, where individuals can choose multiple health insurance schemes based on unobservable and time-varying characteristics. In order to deal with the potential bias error, we chose to control the fixed effects at the individual household level. To avoid the bias error potentially introduced by the self-selection problem and non-linear models (32), we opted for the linear probability model as the identification method. The linear probability model is easier to interpret and faster to run than other logistic models, which is especially important when dealing with large data sets or complex models (33). We use dynamic DID to achieve the dynamic effect of staggered DID and to test the critical parallel pre-trend hypothesis, as shown in Eq. (4). We set the event window period of dynamic DID as three periods before and after the integration policy implementation. Since the CHARLS sample period is generally from July or August of the interview year to July or August of the previous year, many pilot cities implement the urban–rural health insurance integration policy at the end of the calendar year. It is likely that the policy will not work temporarily in the year of the integration policy implementation, but will work in the first period after the year. Therefore, we used the current period as the baseline control group in the dynamic test to avoid the dummy variable trap.


(5) $$\text{Labor}\_\text{suppl}y_{\text{ist}}=\alpha_{0}+\alpha_{1}\text{Dpolic}y_{\text{ist}}+\gamma X_{\text{ist}}+\mu_{i}+\lambda_{t}+\varepsilon_{\text{ist}}$$

(6) $$\begin{array}{l} \text{Poverty}:\text{vulnerablit}y_{\text{ist}}=\delta_{0}+\delta_{1}\text{Dpolic}y_{\text{ist}}+\delta_{2}\text{Labor}:\text{suppl}y_{\text{ist}}\\ \;+\gamma X_{\text{ist}}+\mu_{i}+\lambda_{t}+\varepsilon_{\text{ist}} \end{array}$$

(7) $$\text{Physical}\_\text{examinatio}n_{\text{ist}}=\eta_{0}+\eta_{1}\text{Dpolic}y_{\text{ist}}+\gamma X_{\text{ist}}+\mu_{i}+\lambda_{t}+\varepsilon_{\text{ist}}$$

(8) $$\begin{array}{l} \text{Poverty}\_\text{vulnerablit}y_{\text{ist}}=\kappa_{0}+\kappa_{1}\text{Dpolic}y_{\text{ist}}\\ \;+\kappa_{2}\text{Physical}\_\text{examinatio}n_{\text{ist}}\\ \;+\gamma X_{\text{ist}}+\mu_{i}+\lambda_{t}+\varepsilon_{\text{ist}} \end{array}$$

**Figure 3:**
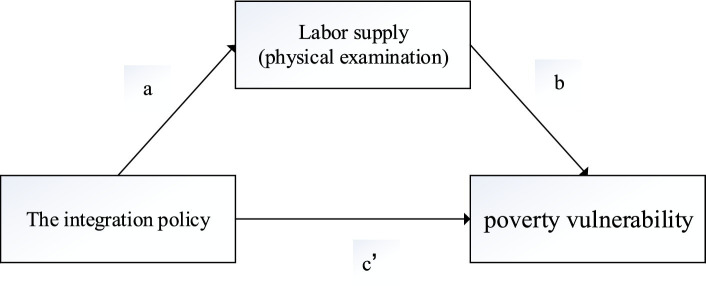
Labor supply and physical examination as the mediator.

As is shown in Figure 3, we used the mediation effect model to test that whether labor supply and physical examinations are important influencing mechanisms for the urban–rural health insurance integration policy to indirectly reduce the poverty vulnerability of rural residents, as shown in Eqs. (5–8) (34, 35).

## 3 Empirical results

### 3.1 Baseline regression results

We report the main estimation results regarding the impact of the urban–rural health integration policy on rural residents’ poverty vulnerability in Table 4, based on Eq. (2). Considering that the CHARLS has only published party membership and ethnic minority data in two data waves (2013 and 2018) and community (or village) characteristics in one data wave (2011), we matched data information across years while assuming that these individual and community characteristics remain stable in recent years. Controlling for these variables in Table 4 (column 2 in Table 4) does not affect the estimation of the integration policy effect. The estimated results show that the urban–rural health insurance integration policy significantly reduces the poverty vulnerability of rural residents by 6.32% (*p* < 0.01). Both the OSOS and OSMS can greatly alleviate chronic poverty. However, the policy effect for alleviating chronic poverty of the OSOS (6.27%, *p* < 0.01) is greater than that of the OSMS (3.25%, *p* < 0.01).

**Table 4:** The impact of urban–rural health insurance integration on poverty vulnerability.

Variable	Vulnerability	Vulnerability	Vulnerability
	(1)	(2)	(3)
*Dpolicy*_ist_	−0.0696***	−0.0632***	
	(0.0141)	(0.0137)	
*Dpolicy*_ist, OSOS_			−0.0627***
			(0.0117)
*Dpolicy*_ist,OSMS_			−0.0325**
			(0.0149)
Age	−0.0058***	−0.0054***	−0.0054***
	(0.0003)	(0.0003)	(0.0003)
Gender	−0.0870***	−0.0838***	−0.0838***
	(0.0054)	(0.0053)	(0.0053)
Marital status	−0.2366***	−0.2452***	−0.2451***
	(0.0092)	(0.0088)	(0.0088)
Education level	−0.0455***	−0.0429***	−0.0429***
	(0.0008)	(0.0008)	(0.0008)
Party membership		−0.0289***	−0.0287***
		(0.0097)	(0.0097)
Ethnic minority		−0.0939***	−0.0927***
		(0.0108)	(0.0108)
Self-assessed health	−0.0828***	−0.0726***	−0.0725***
	(0.0058)	(0.0057)	(0.0057)
Family size	0.0087***	0.0109***	0.0109***
	(0.0019)	(0.0019)	(0.0019)
ln(household income)	−0.0366***	−0.0305***	−0.0306***
	(0.0019)	(0.0018)	(0.0018)
Eligibility for social assistance	0.0499***	0.0454***	0.0455***
	(0.0089)	(0.0086)	(0.0086)
Asphalt roads		−0.0793***	−0.0788***
		(0.0060)	(0.0060)
Sewer system		−0.1928***	−0.1940***
		(0.0066)	(0.0066)
ln(*per capita* GDP)	−0.1338***	−0.0918***	−0.0895***
	(0.0050)	(0.0051)	(0.0052)
Medical beds	−0.0025***	−0.0051***	−0.0054***
	(0.0005)	(0.0005)	(0.0005)
Observations	33,452	33,452	33,452
Adj. *R*^2^	0.6035	0.6268	0.6269

Standard errors are in parentheses. The statistical significance is as follows: *** denotes *p* < 0.01; ** denotes *p* < 0.05; and * denotes *p* < 0.1. We controlled all estimation results for individual and year fixed effects.

### 3.2 Heterogeneity analysis

To understand the different policy effects on various populations in terms of health and expense across two dimensions, we conducted a heterogeneity analysis and explore the potential mechanism. We chose self-assessed health for subjective health and chronic disease for objective health as a health dimension to group the samples. Integration policies are more likely to reduce the poverty vulnerability of groups with relatively poor health (7.84%, *p* < 0.1) than those with fairly good health (6.07%, *p* < 0.01). They also significantly reduce the poverty vulnerability of the group with chronic diseases by 9.59% (*p* < 0.01). We grouped the samples by household consumption and medical expenditure as expense dimensions. The integration policy can significantly reduce the poverty vulnerability of the low consumption and low medical expenditure groups by 8.6% (*p* < 0.01) and 7.64% (*p* < 0.01), respectively, compared to their counterparts. The heterogeneity analysis shows that the urban–rural health insurance integration policy has a greater effect on groups with poor health and low expense levels. We also conducted a preliminary exploration of the mechanism through heterogeneity analysis. The integration can significantly reduce household medical expenditure and has a greater effect on the low-consumption group (50.18%, *p* < 0.01) (Table 5).

**Table 5:** Heterogeneous impact of urban–rural integration on poverty vulnerability.

Variable	Vulnerability	Vulnerability	Vulnerability	Vulnerability	Vulnerability
	Good health	Poor health	Chronic disease	No chronic disease	Low household consumption
	(1)	(2)	(3)	(4)	(5)
*Dpolicy*_ist_	−0.0607***	−0.0784*	−0.0959***	−0.0377	−0.0860***
	(0.0193)	(0.0419)	(0.0219)	(0.0305)	(0.0253)
Control	Y	Y	Y	Y	Y
Observations	19,768	3,873	14,957	6,954	12,374
Adj. *R*^2^	0.6514	0.6178	0.6602	0.6714	0.7056

Variable	Vulnerability	Vulnerability	Vulnerability	ln(medical expenditure)	ln(medical expenditure)
	High household consumption	Low medical expenditure	High medical expenditure	Low household consumption	High household consumption
	(6)	(7)	(8)	(9)	(10)
*Dpolicy*_ist_	−0.0195	−0.0764***	−0.0529**	−0.5019***	−0.4148**
	(0.0205)	(0.0292)	(0.0227)	(0.1845)	(0.2097)
Control	Y	Y	Y	Y	Y
Observations	9,498	8,359	13,741	13,431	10,912
Adj. *R*^2^	0.6111	0.682	0.6504	0.474	0.491

Standard errors are in parentheses. The statistical significance is shown as follow: *** denotes *p* < 0.001; ** denotes *p* < 0.01; and * denotes *p* < 0.05. The grouping basis of the heterogeneity test is the median of household consumption and medical expenditure. The dependent variable for columns (9) and (10) is the logarithm of the family’s medical expenditure.

### 3.3 Mechanisms

We employed the mediation model to explore two important mechanism channels, labor supply and physical examination, which have not been paid attention to in previous literature. The mediation model was set according to Eqs. (2, 5, 6) for labor supply and Eqs. (2, 7, 8) for physical examination. We report the estimation results of mediation analyses in Table 6. For labor supply and physical examination, the total effect (path c) of integration policy is significantly negative according to the estimation for Eq. (2). The coefficient of path a ( $\alpha_{1}$ =14.34%, *p* < 0.01, $\eta_{1}$ =6.28%, *p* < 0.01) is significantly positive in column (1, 3), and the coefficient of path b ( $\delta_{2}$ =0.95%, p < 0.01, $\kappa_{2}$ =2.09%, *p* < 0.01) is significantly negative in column (2, 4). The estimation of the mediation effect model showed that the indirect effects of labor supply and physical examination were 0.14%, 0.13% ( $\alpha_{1}\times \delta_{2}$ , $\eta_{1}\times \kappa_{2}$ ), respectively. The proportion of indirect effect to total effect is 2.16, 2.05% $(\frac{\alpha_{1}\times \delta_{2}}{\alpha_{1}\times \delta_{2}+\delta_{1}}$ , $\frac{\eta_{1}\times \kappa_{2}}{\eta_{1}\times \kappa_{2}+\kappa_{1}})$ .

**Table 6:** Mechanism analysis of the urban–rural health insurance integration.

Variable	Labor supply	Vulnerability	Physical examination	Vulnerability
	(1)	(2)	(3)	(4)
*Dpolicy*_ist_	0.1434***	−0.0626***	0.0628***	−0.0627***
	(0.0251)	(0.0139)	(0.0146)	(0.0139)
Mediation variable		−0.0095***		−0.0209***
		(0.0036)		(0.0060)
Age	−0.0139***	−0.0053***	0.0073***	−0.0050***
	(0.0005)	(0.0003)	(0.0003)	(0.0003)
Gender	−0.1335***	−0.0857***	0.0290***	−0.0837***
	(0.0093)	(0.0054)	(0.0057)	(0.0053)
Marital status	−0.0509***	−0.2456***	0.0035	−0.2448***
	(0.0130)	(0.0090)	(0.0094)	(0.0089)
Education level	0.0196***	−0.0428***	0.0045***	−0.0429***
	(0.0016)	(0.0009)	(0.0010)	(0.0009)
Party membership	0.1070***	−0.0280***	0.0600***	−0.0277***
	(0.0196)	(0.0099)	(0.0114)	(0.0099)
Ethnic minority	−0.0595***	−0.0935***	−0.0316***	−0.0935***
	(0.0164)	(0.0109)	(0.0115)	(0.0109)
Self-assessed health	0.1108***	−0.0773***	−0.0058	−0.0782***
	(0.0111)	(0.0059)	(0.0066)	(0.0059)
Social activity	−0.0111**	0.0039	0.0239***	0.0045*
	(0.0051)	(0.0027)	(0.0031)	(0.0027)
Chronic disease	−0.0681***	−0.0241***	0.0583***	−0.0222***
	(0.0099)	(0.0057)	(0.0061)	(0.0057)
Family size	−0.0256***	0.0107***	−0.0078***	0.0108***
	(0.0031)	(0.0019)	(0.0020)	(0.0019)
ln(household income)	0.0397***	−0.0294***	0.0081***	−0.0298***
	(0.0017)	(0.0019)	(0.0011)	(0.0019)
Eligibility for social assistance	−0.0656***	0.0457***	0.0144	0.0465***
	(0.0127)	(0.0087)	(0.0093)	(0.0087)
Asphalt roads	0.0232**	−0.0815***	0.0123*	−0.0812***
	(0.0103)	(0.0061)	(0.0064)	(0.0061)
Sewer system	0.0685***	−0.1915***	0.0507***	−0.1911***
	(0.0134)	(0.0067)	(0.0077)	(0.0067)
ln(*per capita* GDP)	0.0824***	−0.0921***	0.0482***	−0.0919***
	(0.0091)	(0.0052)	(0.0056)	(0.0051)
Medical beds	−0.0048***	−0.0050***	0.0024***	−0.0050***
	(0.0009)	(0.0005)	(0.0005)	(0.0005)
Observations	38,103	32,637	38,129	32,654
R-squared	0.4686	0.6281	0.5455	0.6283

Standard errors are in parentheses. The statistical significance is shown as follow: *** denotes *p* < 0.001; ** denotes *p* < 0.01; and * denotes *p* < 0.05. The labor supply variable was set as the logarithm of the number of months worked in the past year. The physical examination variable was set as whether physical examination had been undertaken since the last interview. Column (2) in Table 4 and columns (1–2) in Table 6 jointly give the estimation results of the labor supply for the mediation model, corresponding to models (2), (5), and (6), respectively. Column (2) in Table 4 and columns (3–4) in Table 6 jointly give the estimation results of the physical examination for the intermediary effect model, corresponding to models (2), (7), and (8), respectively.

### 3.4 The parallel trend assumption test and robustness check

We report the estimation results of dynamic DID and robustness tests in Table 7. The interview time of the CHARLS samples is mainly concentrated in summers, from July or August of the survey year to July or August of the previous year. However, most pilot cities implement the urban–rural health insurance integration at the end of the year, which has a potential possibility that the policy effect will not work immediately. There may be a policy lag effect on the model estimation results. Therefore, the current period was set to the baseline control group in the dynamic DID model to avoid the dummy variable problem in our study. The estimation of dynamic DID indicates that there is no significant policy effect three periods before the implementation of the integration policy, and the parallel trend test is satisfied. The effect of the integration policy has significantly reduced the poverty vulnerability of rural residents after the implementation of three periods, and the effect of the policy has begun to decline as time goes by. If staggered DID includes the treatment groups (early treatment groups) from the beginning of the sample period, this could raise potential bias issues (25). The Chinese government promoted the urban–rural health insurance integration in 2016, and most prefecture-level cities began implementing the integration policy after 2017. We adopted a more flexible research design by excluding the CHARLS data of 2018 to conduct the robustness test. The estimated regression results show that there is no serious bias issue in Table 7, column (2). We recalculated poverty vulnerability using the World Bank poverty standard of $1.25 per person per day in Table 7, column (2), and the results remain robust with China’s national poverty line estimation result. We estimated the results of the placebo test by moving forward the policy implementation time for all prefecture-level cities by one period, and the estimation result in Table 7, column (4), indicates that the placebo test is satisfied.

**Table 7:** The estimation of dynamic DID and robustness check.

Variable	Vulnerability	Vulnerability	Vulnerability	Vulnerability
	(1)	(2)	(3)	(4)
*Dpolicy*_ist_		−0.0605***	−0.0586***	
		(0.018)	(0.014)	
*Dpolicy*_ist_(placebo)				0.0122
				(0.011)
*Dpolicy*_is,t-3_	−0.0143			
	(0.009)			
*Dpolicy*_is,t-2_	−0.0090			
	(0.013)			
*Dpolicy*_is,t-1_	−0.0251			
	(0.016)			
*Dpolicy*_is,t + 1_	−0.1149***			
	(0.013)			
*Dpolicy*_is,t + 2_	−0.1005***			
	(0.017)			
*Dpolicy*_is,t + 3_	−0.0824***			
	(0.016)			
Control	Y	Y	Y	Y
Observations	33,452	18,034	33,452	33,452
Adj. *R*^2^	0.628	0.665	0.646	0.626

Standard errors are in parentheses. The statistical significance is shown as follows: *** denotes *p* < 0.001; ** denotes *p* < 0.01; and * denotes *p* < 0.05. The estimation results in column (1) of Table 7 is according to model (4). Column (2) in Table 7 reports the robustness estimation results according to model (2) after excluding the CHARLS 2018 sample.

## 4 Discussion

### 4.1 The policy implications of the baseline regression evaluation

We examined the impact of the urban–rural health insurance integration policy on the long-term poverty of rural residents. We found that the urban–rural health insurance integration policy significantly reduced poverty vulnerability, and the OSOS was more effective than the OSMS. This difference of the policy effect indicates very critical policy implications. The implementation of a comprehensive unified medical insurance scheme policy in the OSOS from the very beginning can fully meet the medical demand of rural residents. As previous studies have shown (15, 17), urban–rural health insurance integration policies promote the accessibility of healthcare services, which can repair the current health human capital of rural residents in a timely manner and reduce the probability of poverty in the future period. The OSOS treats all urban and rural residents equally for the same contribution and benefits. The OSOS is the integration of urban and rural health insurance systems in real practice. The OSMS stabilizes the coverage of health insurance by providing multiple schemes for rural residents, preventing the probability of low- and middle-income rural residents leaving the health insurance system. But the OSMS is still in essence a transitional health insurance system arrangement that does not really address the separation between urban and rural in health insurance and does not fully promote health insurance equity. The unified integration policy can better achieve the effect of reducing long-term poverty. Therefore, considering the fairness of health insurance from the perspective of policy practice will be a policy design direction to be considered in the future reform of China’s health insurance system. China’s policy experience has important lessons for developing countries that continue to improve their health insurance systems.

### 4.2 Heterogeneity analysis indicates that integration policy improves the benefits for vulnerable groups

Previous literature on the evaluation of the policy effect of China’s basic medical insurance showed that although the NCMS significantly improved the healthcare utilization for rural residents, it did not significantly reduce the out-of-pocket payments for rural residents (8, 9, 12). The URBMI for urban residents has more significant policy effects, which not only can significantly reduce the medical burden of urban residents but has a significant health promotion effect. However, the rich benefit more from health insurance funding of the URBMI than the poor (13, 15). According to the heterogeneity analysis in our study, the URRBMI merged from the NCMS and URRBMI can significantly reduce poverty vulnerability for low-consumption and poor health level groups, and can better reduce medical expenditure for low-consumption groups. First, the urban–rural health insurance integration policy was designed to promote equity in health insurance by increasing the healthcare reimbursement benefits for rural residents without significantly increasing the cost of participation. Second, the implementation of the integration policy has expanded the catalog of medical services and medicines. These policy initiatives have enhanced the accessibility of healthcare services for vulnerable groups. Third, the integration policy has elevated the level of health insurance financing from county to prefecture-level cities. The low-income groups will not be deprived of the economic protection provided by health insurance because of the administrative geographical location. It provides further key policy evidence that promoting health insurance equity can benefit vulnerable groups.

### 4.3 Mechanism analysis

Previous studies have explored the mechanisms for the impact of the urban–rural health insurance integration especially using the reimbursement channels of rural residents’ inpatient care utilization (17–19). The implementation of the urban–rural medical insurance integration has provided equitable health insurance treatment for rural residents by narrowing the reimbursement gap between urban and rural areas. The catalog of medicines and the scope of healthcare services have been greatly expanded. Rural residents have fully meet their demand for healthcare utilization, improving the under-treatment situation. Village (or community) health centers that provide primary healthcare to rural residents have higher reimbursement rates after the integration policy.

The urban–rural health insurance integration promotes the financial pooling of the NCMS from county level to prefecture-level cities and effectively alleviates the “lock-in” effect by the county-level finance pooling of the NCMS on rural employment. Improving the portability of health insurance greatly increases the possibility for rural residents to seek healthcare services across urban and rural areas, and the mobility of rural residents for looking for a job with much better employment opportunities and higher income in cities. The urban–rural health insurance integration will be more likely to increase the labor supply of rural residents in cities indirectly and eventually reduces the possibility of future poverty among rural residents.

The urban–rural health insurance integration may also change rural families’ expectations for future health investment, producing an investment incentive effect in advance. It will increase physical examinations to meet the actual demand for healthcare service utilization, and rural residents can recover the loss of health human capital in a timely manner. Therefore, the integration policy indirectly increases the labor supply of rural residents to enhance the resilience of rural residents to get out of poverty in the future, and alleviate long-term poverty.

### 4.4 Contributions and limitations

Our findings provide meaningful marginal contributions as follows: first, compared to previous studies, we have distinguished the differences in the role of the OSOS and OSMS in evaluating the effectiveness of the urban–rural health insurance integration policy. Second, heterogeneity analysis indicates that the urban–rural health insurance integration policy achieves the most important policy goal of health equity, allowing vulnerable groups to benefit more from the social health insurance system. The URRBMI merged from the NCMS and URBMI greatly improves the poverty alleviation effects. This has not been further explored in previous studies (17–19, 36). Third, based on a previous paper, we carefully evaluated the policy by controlling for individual fixed effects rather than regional fixed effects, and explored the potential bias issues of staggered DID through a more concise research design. We also provide the dynamic effects of policy evaluation through dynamic DID.

Our paper also has some limitations. First, the impact of urban-rural health insurance integration policy on poverty vulnerability through indirect channels such as labor supply and physical examination, but the policy effects of the indirect channel are very weak in absolute terms. Further research is needed to verify whether there are some other impact channels. Second, in order to assess the difference in policy effects between the OSOS and OSMS for robust outcomes, we used a linear probability model for estimation and identification. Third, the CHARLS only published community-level data in 2011, and there may be data bias when matching to other years.

## 5 Conclusion

In this paper, we examined the impact of the urban–rural health insurance integration policy on the long-term poverty of rural residents. We found that the urban–rural health insurance integration policy significantly reduced poverty vulnerability, and the OSOS is more effective than the OSMS. The urban–rural health insurance integration policy can significantly reduce poverty vulnerability for low-consumption and poor health level groups, and can better reduce medical expenditure for low-consumption groups. Labor supply and physical examination are indirect channels. Our study confirms that the urban–rural health insurance integration achieves the policy effect of reducing the poverty vulnerability of rural residents. As policy implementation continues to improve, the OSMS will gradually shift to the OSOS, and we believe that integrating policies to alleviate poverty is becoming increasingly critical. Promoting equity in the health insurance system remains a powerful and important policy option to alleviate poverty, especially for vulnerable groups.

## Data availability statement

Publicly available datasets were analyzed in this study. This data can be found here: http://charls.pku.edu.cn/.

## Author contributions

ZL: Conceptualization, Data curation, Formal analysis, Funding acquisition, Methodology, Project administration, Software, Supervision, Writing – original draft, Writing – review & editing. YC: Conceptualization, Data curation, Formal analysis, Methodology, Resources, Software, Writing – original draft, Writing – review & editing. JD: Conceptualization, Data curation, Formal analysis, Methodology, Resources, Software, Writing – review & editing.
