# Supplementary material for: Impact of health insurance equity on poverty vulnerability: evidence from urban–rural health insurance integration in rural China
Source: Front Public Health. 2023 Nov 30;11:1328265. doi: 10.3389/fpubh.2023.1328265 (PMC10722085; doi:10.3389/fpubh.2023.1328265)
Supplement: Supplementary file 1 [file Table_1.DOCX]

# Appendix A

Poverty vulnerability of the cross-section data for each period is estimated, and then all the results are integrated to form panel data as the dependent variable of staggered DID model to estimate the integration policy effect. We estimate income equation of the household i given by :

| ${lnincome}_{i}=X_{i}\beta+e_{i}$ | (A.1) |
| --- | --- |

where ${income}_{i}$ is per capita household income, $X_{i}$ is a bundle of household characteristics variables, such as age, education level, gender, party membership, ethnic minority, self-assessed health, chronic disease status, family size, family wage income share, family income expenditure, eligibility for social assistance. $\beta$ is a vector of parameters, and $e_{i}$ is a disturbance term. We estimate the variance equation by assuming that the variance of $e_{i}$ is given by:

| $\sigma_{e,i}^{2}=X_{i}\vartheta$ | (A.2) |
| --- | --- |

A three-step feasible generalized least squares (FGLS) procedure is adopted to estimating the parameters of $\beta$ and $\vartheta$ (29).

| $\hat{e}_{OLS,i}^{2}=X_{i}\vartheta+\varepsilon_{i}$ | (A.3) |
| --- | --- |

First, We get the residual from estimating equation (A.3) by an ordinary least squares (OLS) procedure. The predictions of the residual are used as the weight for asymptotically efficient estimation of the parameter,$\hat{\vartheta}_{FGLS}$ in equation (A.4) as follows:

| $\frac{\hat{e}_{OLS,i}^{2}}{{X_{i}\hat{\vartheta}}_{OLS}}=\left( \frac{X_{i}}{{X_{i}\hat{\vartheta}}_{OLS}} \right)\vartheta+\frac{\varepsilon_{i}}{{X_{i}\hat{\vartheta}}_{OLS}}$ | (A.4) |
| --- | --- |

The consistent estimation of the variance $\sigma_{e,i}^{2}$ is ${X_{i}\hat{\vartheta}}_{FGLS}$. $\hat{\sigma}_{e,i}$is used as critical weight for the eanalysisof the coefficient $\hat{\beta}_{FGLS}$ by FGLS procedure in equation (A.6). Naturally we obtain the expectation and variance of per capita household income in equation (A.7) and (A.8). We can calculate the probability that household i will fall below the poverty line, based on the cumulative density of the standard normal in equation (A.9).

| $\hat{\sigma}_{e,i}=\sqrt{{X_{i}\hat{\vartheta}}_{FGLS}}$ | (A.5) |
| --- | --- |

| $\frac{{lnincome}_{i}}{\hat{\sigma}_{e,i}}=\left( \frac{X_{i}}{\hat{\sigma}_{e,i}} \right)\beta+\frac{e_{i}}{\hat{\sigma}_{e,i}}$ | (A.6) |
| --- | --- |

| $\hat{E}[{lnincome}_{i}\vert X_{i}]=X_{i}\hat{\beta}_{FGLS}$ | (A.7) |
| --- | --- |

| $\hat{V}[{lnincome}_{i}\vert X_{i}]=\hat{\sigma}_{e,i}^{2}{=X_{i}\hat{\vartheta}}_{FGLS}$ | (A.8) |
| --- | --- |

| $\hat{V}_{i,t}=\hat{Pr}[{lnincome}_{i}<lnz\vert X_{i}]=\phi\left( \frac{lnz-{=X_{i}\hat{\vartheta}}_{FGLS}}{\sqrt{{X_{i}\hat{\vartheta}}_{FGLS}}} \right)$ | (A.9) |
| --- | --- |

**Table A1.** Enrolment premium of OSMS for URRBMI in Qiqihar City, Heilongjiang Province

| Scheme | Premium Composition | | |
| --- | --- | --- | --- |
|  | Total Contributions | Individual Contribution | Government Subsidy |
| I | 570 | 150 | 420 |
| II | 700 | 280 | 420 |
| III | 800 | 380 | 420 |

**Table A2.** Benefits package of OSMS for URRBMI in Qiqihar City, Heilongjiang Province

| Benefits package | Inpatient Reimbursement Standard | | | |
| --- | --- | --- | --- | --- |
|  | Township level designated health centers | | Primary designated medical institutions and community health service centers | |
|  | deductible | reimbursement ratio | deductible | reimbursement ratio |
| I | 100 | 90% | 200 | 80% |
| II | 100 | 90% | 200 | 85% |
| III | 100 | 90% | 200 | 85% |
| Benefits package | Secondary designated medical institutions | | Tertiary designated medical institution | |
|  | deductible | reimbursement ratio | deductible | reimbursement ratio |
| I | 300 | 70% | 500 | 60% |
| II | 300 | 75% | 500 | 70% |
| III | 300 | 75% | 500 | 70% |
| Benefits package | Outpatient Reimbursement Standard | | | |
|  | Township level designated health centers | | Primary designated medical institutions and community health service centers | |
|  | deductible | reimbursement ratio | deductible | reimbursement ratio |
| I | 0 | 60% | 50 | 60% |
| II | 0 | 60% | 50 | 60% |
| III | 0 | 60% | 50 | 60% |
| Benefits package | Secondary designated medical institutions | | Tertiary designated medical institution | |
|  | deductible | reimbursement ratio | deductible | reimbursement ratio |
| I | 50 | 60% | 50 | 60% |
| II | 50 | 60% | 50 | 60% |
| III | 50 | 60% | 50 | 60% |
| Benefits package | Inpatient(Outpatient) Reimbursement Ceilings | | | |
| I | 100000(150) | | | |
| II | 150000(150) | | | |
| III | 150000(150) | | | |

**Source:** Notice of the General Office of Qiqihar Municipal People's Government on Printing and Distributing the Administrative Measures of Basic Medical Insurance for Urban and Rural Residents in Qiqihar.(http://www.qqhr.gov.cn/News_showGkmlNews.action?messagekey=111916)

**Notes:** Vulnerable groups participate URRBMI adopt lower premiums. 100 yuan per year for students and children with severe disabilities; 120 yuan per year for severely disabled individuals who have lost their ability to work, as well as elderly people aged 60 and above from low-income families; 120 yuan per year for various types of school students and non school personnel under the age of 18; 160 yuan per year for the minimum income security personnel and rural special poverty support personnel registered by the Official Welfare Department. Vulnerable groups adopt Benefits package II.
